# A Sad Case

**Published:** 1907-02-09

**Authors:** 


### A SAD CASE.

THE Church Army makes a strong appeal for help in the following sad case: The widow of a physician (M.R.C.P. and M.R.C.S.) formerly in excellent practice in the West End of London finds herself in her old age reduced to less than bare necessaries. All she has to depend upon is an annuity of £20 a year from a medical charity (which is sufficient to pay her rent) and 5s. a week given by her children, which is all they can possibly afford. The husband was compelled to relinquish his practice in consequence of a long and painful illness. Unfortunate speculations completed his ruin, and he was unable to provide for his widow and family. The poor old lady, who is eighty-one years of age, is crippled with rheumatism in both knees, and is only able to crawl about a very little. Her sight is very bad, and she is entirely unfit to do any sort of work. It is desired to raise a small fund out of which an allowance can be made to supplement her income. Communications should be addressed to Mr. Colin F. Campbell, Hon. Social Secretary of the Church Army, 55 Bryanston Street, W., who will gladly answer all inquiries.

